# The miR-378c-Samd1 circuit promotes phenotypic modulation of vascular smooth muscle cells and foam cells formation in atherosclerosis lesions

**DOI:** 10.1038/s41598-021-89981-z

**Published:** 2021-05-18

**Authors:** Shengya Tian, Yang Cao, Jinliang Wang, Yongjun Bi, Jingquan Zhong, Xiangbin Meng, Wenyu Sun, Ruixue Yang, Luping Gan, Xuping Wang, Hongshi Li, Rong Wang

**Affiliations:** 1 The Key Laboratory of Cardiovascular Remodeling and Function Research, Chinese Ministry of Education, Chinese National Health Commission and Chinese Academy of Medical Sciences, The State and Shandong Province Joint Key Laboratory of Translational Cardiovascular Medicine, Department of Cardiology, Qilu Hospital, Cheeloo College of Medicine, Shandong University, 107 Wenhuaxi Road, Jinan, 250012 Shandong China; 2 Department of Cardiology, Boston Children’s Hospital, Harvard Medical School, 300 Longwood Avenue, Boston, MA 02115 USA; 3 Division of Hematology and Oncology, Beth Israel Deaconess Medical Center (BIDMC) and Harvard Medical School (HMS), Boston, MA 02215 USA; 4 Department of Medicine/Division of Cardiology, University of California, Los Angeles, CA USA; 5 Department of Biochemistry, University of Utah School of Medicine, Salt Lake City, UT 84112 USA; 6 Clinical Laboratory, Central Hospital of Xinwen Mining Group, 164 Xinkuang Road, Xintai, 271233 Shandong China; 7 Department of Cardiac Surgery, Qilu Hospital, Cheeloo College of Medicine, Shandong University, 107 Wenhuaxi Road, Jinan, 250012 Shandong China; 8 Department of Cardiac Surgery, Qilu Hospital (Qingdao), Cheeloo College of Medicine, Shandong University, 758 Hefei Road, Qingdao, 266035 Shandong China; 9 Department of Anesthesiology, The People’s Hospital of Yichun Jiangxi, Yichun, 336000 Jiangxi China

**Keywords:** Biomarkers, Cardiology, Diseases

## Abstract

MicroRNAs have emerged as key regulators in vascular diseases and are involved in the formation of atherosclerotic lesions. However, the atherosclerotic-specific MicroRNAs and their functional roles in atherosclerosis are unclear. Here, we report that miR-378c protects against atherosclerosis by directly targeting Sterile Alpha Motif Domain Containing 1 (Samd1), a predicted transcriptional repressor. miR-378c was strikingly reduced in atherosclerotic plaques and blood of acute coronary syndrome (ACS) patients relative to healthy controls. Suppression of miR-378c promoted vascular smooth muscle cells (VSMCs) phenotypic transition during atherosclerosis. We also reported for the first time that Samd1 prolonged immobilization of LDL on the VSMCs, thus facilitated LDL oxidation and subsequently foam cell formation. Further, we found that Samd1 contains predicted DNA binding domain and directly binds to DNA regions as a transcriptional repressor. Together, we uncovered a novel mechanism whereby miR-378c-Samd1 circuit participates in two key elements of atherosclerosis, VSMCs phenotypic transition and LDL oxidation. Our results provided a better understanding of atherosclerosis pathophysiology and potential therapeutic management by targeting miR-378c-Samd1 circuit.

## Introduction

Atherosclerosis is a chronic inflammatory disease of the arterial wall and causes a range of complications such as myocardial infarctions, strokes, and peripheral artery disease^1^, ranking the leading cause of morbidity and mortality worldwide. Atherosclerotic lesion formation is a complex process. It’s initiated by endothelial dysfunction and the subendothelial retention of lipoproteins, such as low-density lipoprotein (LDL). The endothelium then becomes activated and secretes chemokines to promote the adhesion of leukocytes and platelets, which is followed by immune cell infiltration and differentiation. Subsequently, macrophages ingest lipids and become foam cells, which is the hallmark of atherosclerosis^2–4^.

Vascular smooth muscle cells (VSMCs) are a major source of plaque cells and present at all stages of atherosclerosis^5^. VSCMs are highly proliferative during embryonic stage of vasculogenesis and constitute the major cells in the media layer of arteries. In adult blood vessels, VSMCs maintain vascular wall function by exhibiting a differentiated and contractile phenotype with non-migratory and non-proliferative ability. In response to vascular injury or atherosclerosis, VSCMs are switched to a synthetic and proliferative phenotype^6^. Activated VSCMs migrate into the intima and contact with endothelial cells (ECs), another major type of vascular cells, to mediate the initiation and development of atherosclerosis^7–9^. Hence, understanding VSCMs phenotypic switch and foam cell formation will provide new sights in atherosclerosis pathophysiology and therapeutic management.

MicroRNAs are 18–22 nucleotides highly conserved single-stranded non-coding RNA molecules that suppress gene expression post-transcriptionally by triggering either translation repression or RNA degradation^10^. MicroRNAs have been found to play essential roles in regulating cellular processes and contribute to numerous diseases progression^11–14^. Recently, accumulating evidence demonstrated that cellular and molecular processes related to the development of atherosclerosis are strongly affected by dysregulation of MicroRNAs^15–17^, including endothelial injury, lipoprotein retention, leukocyte migration, foam cell formation, etc^16^. For example, miR-155 has been found to promote atherosclerosis by repressing Bcl6 in macrophages^18^. miR-155 was upregulated in macrophages in atherosclerotic lesions, which induced CCL2 expression in macrophages via direct suppression of Bcl6. The nuclear factor NF-kB signaling pathway is a critical driver of endothelial dysfunction and atherosclerosis. Studies showed that miR-181a-5p and miR-181a-3p inhibit vascular inflammation by targeting NF-kB signaling pathway and systemic miR-181 delivery reduces NF-kB activity and atherosclerotic lesion formation^19^. However, tissue-specific expression is an important characteristic of MicroRNA^20,21^, different expression profiles in different tissues suggest that MicroRNAs in different tissues may have distinct physiological functions^22,23^. Thus, identifying cell type-specific MicroRNAs and clarifying their biological functions would be useful for understanding the mechanisms of atherosclerosis and developing new therapeutic management.

Here, we investigated the MicroRNAs expression profiling of human normal coronary artery and artery with plaques. Screening of differential MicroRNAs expressions revealed that miR-378c was dramatically suppressed in human arteries with plaques relative to control group. Suppression of miR-378c facilitates the expansion of VSMCs and the formation of foam cells by increasing Sterile Alpha Motif Domain Containing 1 (Samd1). Collectively, our study identified the miR-378c-Samd1circuit that may play a central role in atherogenesis, providing a better understanding of atherosclerosis pathophysiology.

## Materials and methods

### Cell culture

VSMCs were isolated from the thoracic aorta of 4-month-old mice. Cells at passage 3–7 were cultured in low-glucose DMEM (Thermo) supplemented with 10% FBS (Gibco). Quiescence was achieved by serum-starvation in 0.01% serum medium for 48 h before treatments. Proliferation was stimulated with 20 ng/mL platelet-derived growth factor (PDGF). Mouse bone-marrow-derived macrophages (BMDM) were flushed out from femur and seeded in 10% FBS DMEM with M-CSF (50 ng/mL) for 5–7 days. BMDMs were maintained in RPMI 1640 or DMEM medium, supplemented with 10% (v/v) heat-inactivated FCS, 100 U/mL penicillin, 100 mg/mL streptomycin, and 2 mM l-glutamine (Invitrogen).

### Clinical human tissue specimen

Human heart specimens were provided by Tissue Bank at Qilu Hospital of Shandong University. Coronary arteries, included atherosclerotic plaques and normal control vessels, were cut into 0.5 cm pieces and were rapidly stored in liquid nitrogen. Tissue microarray slides used for analysis were constructed by formalin-fixed and then embedded with paraffin. To use these clinical materials for research purposes, prior patients’ written informed consents and approval from Qilu Hospital of Shandong University were obtained, all methods were carried out in accordance with relevant guidelines and regulations. Patient information can be found in the Supplementary Table 5, patient names (and other personally identifiable information) are removed according to the ethical policies.

### MicroRNA microarray analysis

MicroRNA expression was analyzed according to the manufacturer's instructions with the miRCURYTM LNA Array (v.14.0) (Exiqon) provided by Kangchen LTD, Shanghai. Briefly, 100 ng of RNA was labeled with Hy3 or Hy5 fluorescent label and hybridized to the microarray using the MicroRNA Labelling Reagent and Hybridization Kit (Agilent) and Agilent Surehyb chambers^24^. The microarrays were scanned with the Agilent microarray scanner and analyzed with the Agilent Feature Extraction Software and Chipster (Supplementary Table 1).

### Quantitative real-time-PCR

Total RNA was isolated using Trizol followed by DNase (Ambion) treatment, 1 µg total RNA was used to synthesize cDNA using SuperScript III Reverse Transcriptase (Invitrogen). MicroRNAs were reversed transcribed to cDNA using the Reverse Transcription TaqMan MicroRNA Reverse Transcription Kit (Applied Biosystems, 4366597) according to manufacturer’s instructions. qRT-PCR was performed using SYBR Green Master Mix (Vazyme) on a Bio-Rad iCycler^25^. Primer sequences are listed in Supplementary Table 4.

### Protein extraction and western blotting

For total protein collection, cells were harvested and lysed using RIPA buffer (50 mM Tris–HCl, pH 8.0; 150 mM NaCl; 5 mM EDTA; 0.1% SDS; and 1% NP-40) supplemented with protease inhibitor cocktail^26^. For secreted Samd1 collection, the culture medium containing the Samd1 was filtered through a 0.2 μm membrane and then concentrated by protein concentrator (Thermo, 10 K, 88527). Membrane protein extraction was performed with Membrane Protein Extraction Kit (Thermo, 89842) according to manufacturer’s instructions.

The protein concentration was determined by the Bradford assay, and equal amounts of protein in the lysates were boiled and fractionated by 7–12% SDS-PAGE. Primary antibodies against the following proteins were used: ApoB, β-actin, LaminA, Cadherin (Proteintech), Samd1 (Abcam). Signal was detected using Western ECL Substrate (Bio-Rad). To measure signals from the same gel, some full-length original blots might not be able to obtained.

### Plasmid construction

shRNAs in PLKO vector against Samd1 were commercially purchased (Sigma-Aldrich).

### Transfection

100 nM of MicroRNA mimics (Ruibo Company) were transfected into cells in each well of a six-well plate using Lipofectamine-2000. The inhibitor of miR-378c (5′-ACUGGACUUGGAGUCAGAAGC-3′) was a single RNA sequence exactly complementary to miR-378c.

### Dual luciferase reporter assay

Samd1-3′UTR and Samd1-3′UTR-mut were inserted into the pSI-CHECK-2 dual-luciferase reporter vector (Promega), designated as pSI-Samd1-3′UTR and pSI-Samd1-3′UTR-mut. Either pSI-Samd1-3′UTR or pSI-Samd1-3′UTR-mut were co-transfected with miR-378c mimics or CTR into HEK293 using Lipofectamine 2000 (Invitrogen). Firefly luciferase activity and Renilla luciferase activity were measured 48 h after transfection using a Dual Luciferase Reporter Assay System (Promega)^27^.

### Blood sampling and blood miR-378c, Samd1, oxLDL measurement

This study was conducted at Qilu Hospital of Shandong University. The protocols for blood sampling were approved by the local ethics committee of Qilu Hospital of Shandong University. Written informed consent was obtained from all subjects and all methods were carried out in accordance with relevant guidelines and regulations. Patient information can be found in the Supplementary Table 5, patient names (and other personally identifiable information) are removed according to the ethical policies. Venous blood samples (2 mL) were collected before coronary angiography (CAG) in EDTA containing tubes (1.6 mg EDTA/mL blood) and plasma was then isolated by centrifugation (3000 rpm for 10 min at 4 °C).

Blood MicroRNA was isolated using miRNeasy Isolation Kit (Qiagen, 217184) according to manufacturer’s protocol. Blood Samd1 was detected by protein spectrometry. A mAb-4E6 based competition ELISA was used for measuring oxidized LDL in the blood (Mercodia, 10-1143-01) following the manufacturer's instructions.

### Isolation of LDL and measurement of LDL oxidation in vitro

Blood was obtained from healthy volunteers in the presence of 0.01% EDTA. LDL was isolated by density-gradient ultracentrifugation as described before^28^, After the isolation, EDTA existing in LDL was removed by a Sephadex G-25 column (Pharmacia, PD-10) equilibrated with phosphate-buffered saline (PBS). Protein concentration was measured using a Bradford protein assay kit (Bio-Rad).

LDL was diluted (50 μg/mL) and incubated at 37 °C with VSMCs in the presence of 10 μM CuSO_4_ for 48 h to prepare the oxidized LDL (ox-LDL). LDL oxidation was determined by TBARS assay. TBARS assay was performed according to the procedure of Rômulo Pillon Barcelos et al.^29^. The samples were centrifuged (10,000 rpm) at 10 °C for 30 min to remove the pellets, final volume of the supernatants was made up to 2 mL with phosphate buffer (pH 7.4). After incubation, 500 µL of mixture were mixed with 250 µL of TBA (1% in 50 mM of NaOH) and TCA (0.28%). Samples were again incubated at 95 °C for 45 min. After cooling and centrifugation at 2000 rpm (10 min) fluorescence was taken at 532 nm and emission at 600 nm. Quantification of TBARS was measured by comparison with a standard curve of malondialdehyde (MDA) equivalents generated by acid-catalyzed hydrolysis of 1,1,3,3-tetramethoxypropane.

### Secreted pro-inflammatory cytokines detection

Secreted pro-inflammatory cytokines (TNF-α) and interleukin (IL)-6 were detected in aliquots of macrophage supernatants and quantitated by ELISA (R&D Systems). The entire procedure was performed according to the manufacturer’s instructions.

### Samd1 structure prediction

The protein structure prediction tool Phyre2 for homology modelling and through phylogenetic analyses^30^. Predicted models were visualized and analyzed further used by UCSF Chimera (1.15)^31^. Fpocket (2.0) was used to measure the volume of a defined binding pocket^32^. STRING v10 was used to analyze the protein–protein interaction^33^. The mutational sensitivity was analyzed by SuSPect^34^.

### Statistical analysis

The data were presented as mean ± SD or mean ± SEM of at least three independent experiments. P values were calculated by using Student’s t test. Statistical significance was defined as P < 0.05.

## Results

### miR-378c expression is decreased in human atherosclerosis

**Figure 1:**
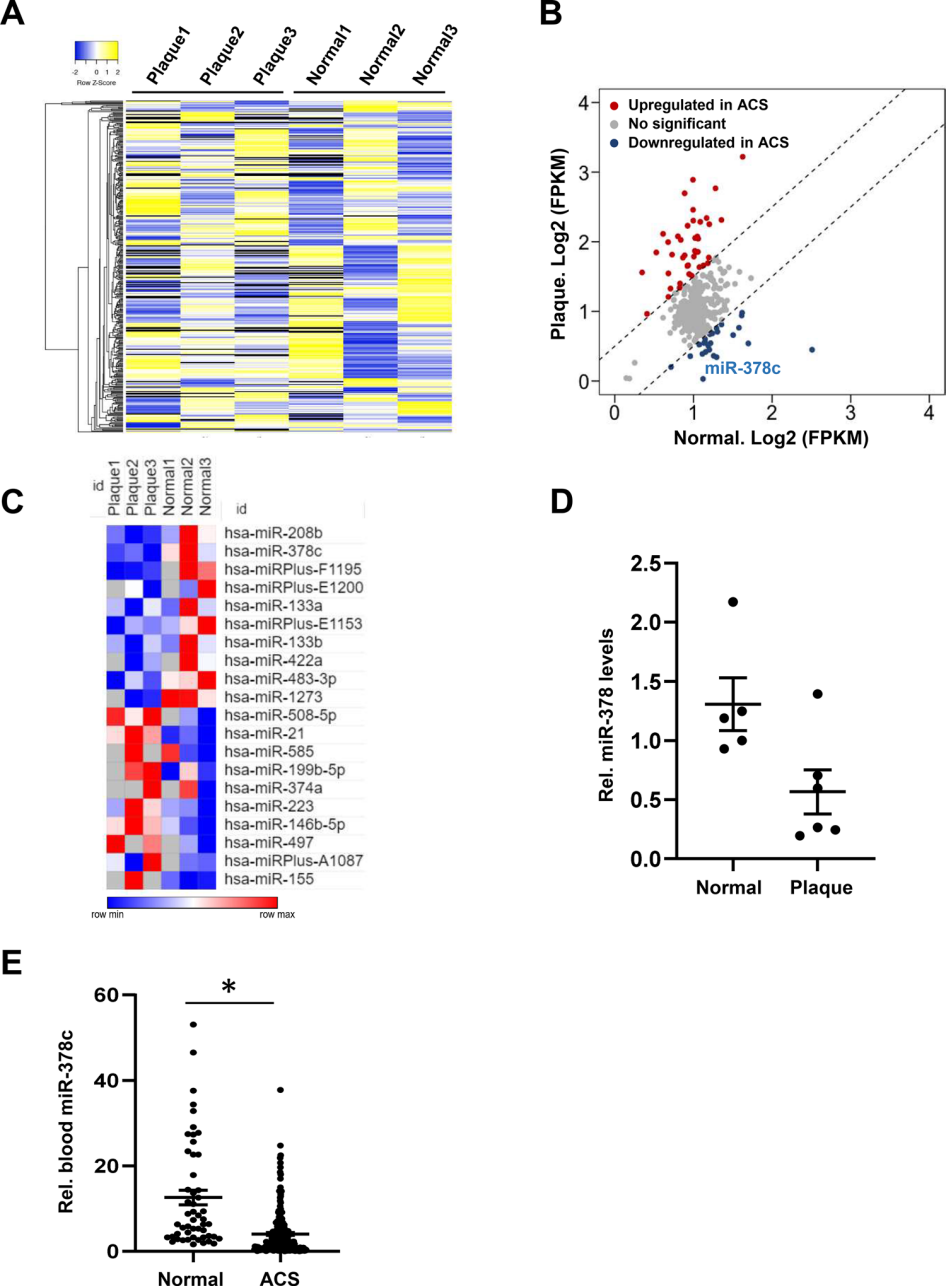
Suppression of miR-378c in human atherosclerosis. (**A**) Heatmap showing MicroRNAs expression profiles in human normal coronary artery and artery with plaques. The colors indicate the ln-transformed FPKM values. (**B**) MicroRNA array analysis for human normal coronary artery and artery with plaques. Each point represents FPKM value for individual MicroRNA. MicroRNAs that were significantly up-regulated and down-regulated in artery with plaques were highlighted in red and blue, respectively. (**C**) Heatmap showing top differentially regulated MicroRNAs (P < 0.05) in human artery with plaques relative to normal controls. (**D**) qRT-PCR analysis of miR-378c expression in atherosclerotic plaques tissues (n = 6) and normal controls (n = 5). Data were presented as mean ± SD. (**E**) Blood miR-378c levels were measured in 215 ACS patients and 52 healthy subjects. Data were presented as mean ± SD. *P < 0.001 as compared between two groups.

To identify novel MicroRNAs that are important for the formation of atherosclerosis, we performed microarray for human normal coronary artery and artery with plaques. The number of blood vessel lesions was positively correlated with fasting blood glucose (FBG) and low-density lipoprotein (LDL) levels and inversely correlated with high-density lipoprotein (HDL) levels (Supplementary Fig. S1), which is consistent with clinical phenotypes^35^. Our microarray data revealed that 34 MicroRNAs were significantly upregulated while 21 MicroRNAs were significantly downregulated in artery with plaques relative to normal controls (Fig. 1A,B, Supplementary Table 2). Among all differential expression MicroRNAs, miR-378c showed the lowest abundance in plaque samples relative to controls (Fig. 1B,C), suggesting a loss of miR-378c in human atherosclerosis.

Human miR-378 family of MicroRNAs (miR-378a-3p/b/c/d/e/f/g/h/i/j) are encoded by different genes but they share identical seed sequences for mRNA targeting. miR-378 family of MicroRNAs have been reported to play an important role in a number of diseases^36,37^. For instance, decreased miR-378 level indicated high tumor invasiveness and poor prognosis in gliomas^38^. miR-378 was also reported to be the most prominent MicroRNA in adult rat cardiomyocytes and regulate cardiac hypertrophy by combined repression of mitogen-activated protein kinase pathway factors^39^. qRT-PCR analysis confirmed the decrease of miR-378c in early atherosclerotic plaques tissues relative to normal controls (Fig. 1D). To further confirm the association between miR-378c expression and atherosclerosis, we measured miR-378c levels in blood samples collected from 215 acute coronary syndromes (ACS) patients and 52 healthy controls. We observed a striking decrease in miRNA-378c levels in ACS patients relative to health controls (Fig. 1E), which is consistent with its lower abundance in atherosclerotic plaques. Taken together, these results demonstrated that miR-378c expression was aberrantly suppressed in atherosclerotic lesions and may play important roles in the formation or progression of atherosclerosis.

### Down-regulation of miR-378c promotes VSMC phenotypic modulation

**Figure 2:**
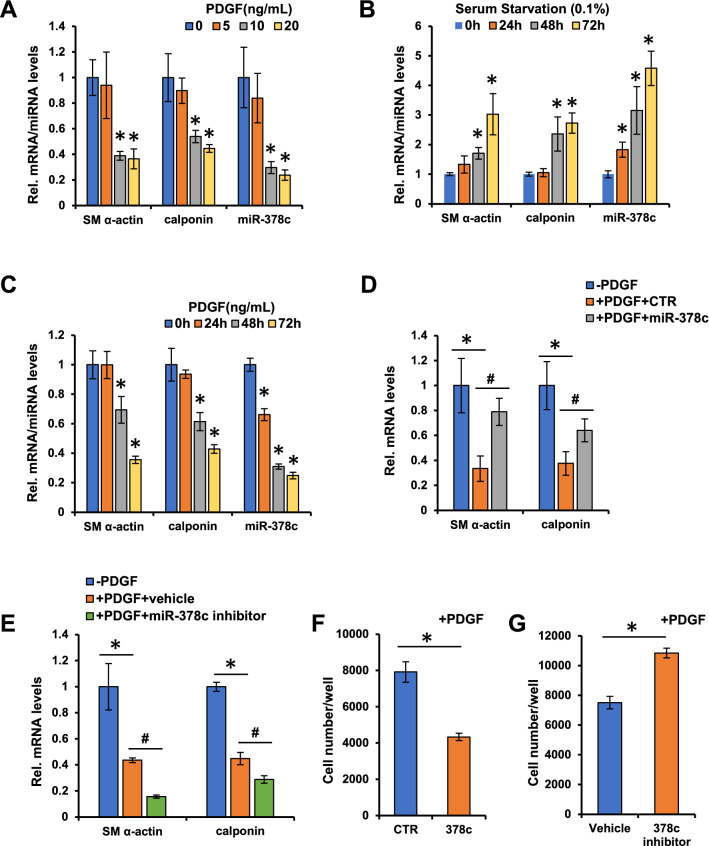
miR-378c suppresses VSMCs phenotypic modulation. (**A**) qRT-PCR analysis of SM α-actin, calponin and miR-378c in VSMCs stimulated with PDGF-BB for 48 h after 24 h serum withdrawal. Data were presented as mean ± SD, n = 4. *P < 0.05 as compared with non-PDGF treated group. (**B**) qRT-PCR analysis of SM α-actin, calponin and miR-378c in serum-starved (0.1%) VSMCs. Data were presented as mean ± SD, n = 4. *P < 0.05 as compared with 0 h group. (**C**) qRT-PCR analysis of SM α-actin, calponin and miR-378c in VSMCs treated with PDGF-BB after 24 h serum withdrawal. Data were presented as mean ± SD, n = 4. *P < 0.05 as compared with 0 h group. (**D**) VSMCs stimulated with PDGF-BB for 48 h were further transfected with miR-378c mimic or mimic control, SM α-actin and calponin were analyzed by qRT–PCR. Data were presented as mean ± SD, n = 4. *P < 0.05 as compared with non-PDGF-treated group; ^#^P < 0.05 as compared with PDGF-treated mimic control group. (**E**) VSMCs stimulated with PDGF-BB were further transfected with miR-378c inhibitor or vehicle (DMSO), VSMC differentiation markers SM α-actin and calponin were analyzed by qRT–PCR. Data were presented as mean ± SD, n = 4. *P < 0.05 as compared with non-PDGF-treated group; ^#^P < 0.05 as compared with PDGF-treated vehicle group. (**F**) VSMCs stimulated with PDGF-BB were further transfected with miR-378c mimic or mimic control, cell numbers were examined. Data were presented as mean ± SD, n = 4. *P < 0.05 as compared with PDGF-treated mimic control group. (**G**) VSMCs stimulated with PDGF-BB were further transfected with miR-378c inhibitor or vehicle (DMSO), cell numbers were examined. Data were presented as mean ± SD, n = 4. *P < 0.05 as compared with PDGF-treated vehicle group.

To investigate the functional role of miR-378c in atherosclerosis, we thus analyzed the expression of miR-378c during the progression of atherosclerotic lesion. Interestingly, we observed down-regulation of miR-378c in the blood of patients with early atherosclerotic lesion, indicating that miR-378c may contribute to the formation of atherosclerosis (data not shown). Vascular smooth muscle cells (VSMCs) are the stromal cells of the vascular wall and involved in the regulation of arterial tone, blood pressure, and blood supply of the tissues^40,41^. The phenotypic transition of VSMCs from the differentiated state to dedifferentiated state is accompanied by cell proliferation and migration, which are hallmarks of the onset of atherosclerosis^42–44^. To test the association between miR-378c and VSMC phenotypic transition, VSMCs were treated with platelet-derived growth factor (PDGF) for 48 h to induce proliferation. Compared with differentiated VSMCs, miR-378c expression levels were suppressed by PDGF treatment in a dose-dependent manner (Fig. 2A). Accordingly, SMC differentiation markers smooth muscle (SM) α-actin and calponin were also reduced by PDGF treatment (Fig. 2A). In addition, VSMCs subjected to serum starvation (0.1%) resulted in an increase of miR-378c in a time-dependent manner, which is accompanied by increased expression of SM α-actin and calponin (Fig. 2B). These results demonstrated that miR-378c was abundant in differentiated VSMCs and decreased upon dedifferentiation (Fig. 2A,B), supporting that miR-378c is associated with phenotypic transition of VSMCs.

Further, we treated VSMCs with PDGF and measured the expression of miR-378c, SM α-actin and calponin at different time points after treatment. miR-378c level was reduced in a time-dependent manner and was significantly reduced after 24 h treatment, which preceded the decrease of SM α-actin and calponin (Fig. 2C), suggesting that miR-378c may contribute to the phenotypic transition. To test the functional role of miR-378c in phenotypic transition, we introduced miR-378c mimic into VSMCs in the presence of PDGF treatment. The decrease of SM α-actin and calponin expression induced by PDGF were restored by miR-378c overexpression (Fig. 2D), suggesting that miR-378c suppressed the dedifferentiation of VSMCs. As expected, miR-378c inhibitor further reduced SM α-actin and calponin expression induced by PDGF (Fig. 2E), which proved our hypothesis that miR-378c suppressed the phenotypic transition of differentiated VSMCs. In addition to the marker genes, miR-378c overexpression decreased while inhibition increased VSMCs cell number (Fig. 2F,G), demonstrating that miR-378c suppresses cell proliferation. Together, miR-378c suppresses the proliferation and phenotypic transition of VSMCs.

### Samd1 is a downstream target of miR-378c involving in atherosclerosis

**Figure 3:**
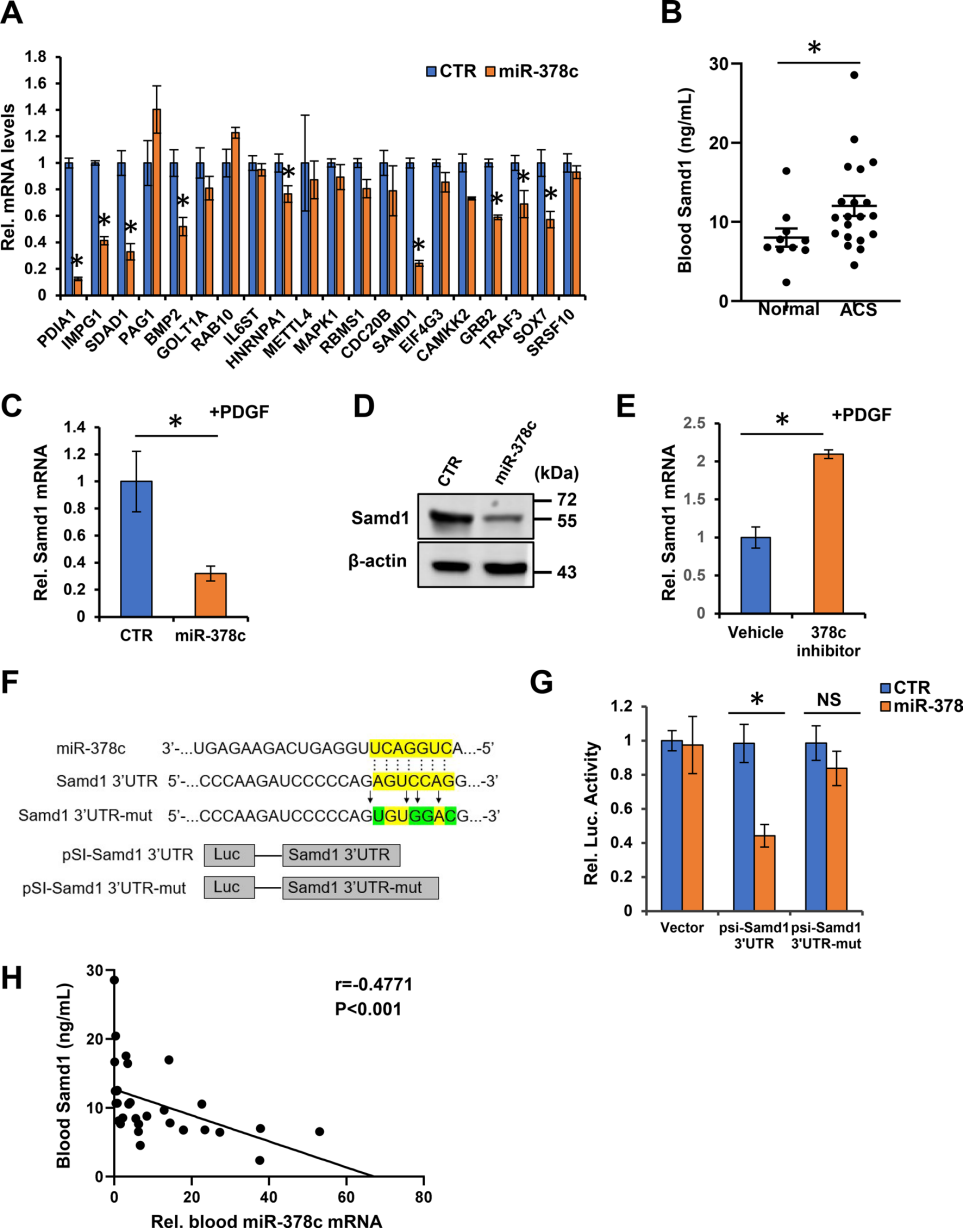
Identification of miR-378 targets in VSMCs. (**A**) Genes contain evolutionary conserved predicted binding sites of human miR-378c and involved in atherosclerosis were assessed by the regulation of miR-378c in VSMCs stimulated with PDGF-BB using qRT-PCR. Data were presented as mean ± SD, n = 4. *P < 0.05 as compared with PDGF-treated mimic control group. (**B**) Blood Samd1 protein levels were measured in 20 ACS patients and 10 healthy subjects. Data were presented as mean ± SD. *P < 0.001 as compared between two groups. (**C**,**D**) VSMCs stimulated with PDGF-BB were further transfected with miR-378c mimic. Samd1 mRNA and protein levels were measured by qRT-PCR (**C**) and western blotting (**D**), respectively. Data were presented as mean ± SD, n = 4. *P < 0.05 as compared with PDGF-treated mimic control group. β-actin serves as a loading control. (**E**) VSMCs stimulated with PDGF-BB were further transfected with miR-378c inhibitor. Samd1 mRNA level was measured by qRT-PCR. Data were presented as mean ± SD, n = 4. *P < 0.05 as compared with PDGF-treated vehicle group. (**F**) Predicted base pairing between miR-378c and 3′UTR of Samd1 by RNA hybrid software. Wild-type or mutated (mut) 3′UTR of Samd1 sequence inserted into dual-luciferase vector were shown. (**G**) miR-378c mimics were co-transfected with pSI-Samd1-3′UTR or pSI-Samd1-3’UTR-mut into HEK293 cells followed by dual-luciferase analysis. *P < 0.05 compared between the indicated groups, n = 4. (**H**) Correlation between blood miR-378c level and blood Samd1 protein level. n = 30. Statistical significance was determined by the chi-square test.

MicroRNAs have been shown to play key roles in modulating cell fate through suppressing the expression of their target genes. We next investigated genes that might be the downstream targets of miR-378c and promote atherosclerosis using two bioinformatic tools, TargetScan and miTALOS^45,46^. By evaluating the evolutionary conserved predicted binding sites of human miR-378c and involvement in atherosclerosis, 20 candidate genes were identified (Fig. 3A). Next, we performed qRT-PCR to measure the effect of miR-378c on these candidates in VSMCs. A number of candidates were suppressed by miR-378c (Fig. 3A). Among them, Samd1 was strikingly suppressed by miR-378c and was predicted to have important function in VSMCs phenotypic transition (Fig. 3A). Moreover, blood Samd1 protein level was much higher in ACS patients relative to healthy controls (Fig. 3B), suggesting its potential contribution in atherosclerotic lesion formation.

Samd1 is a newly identified secreted protein with unknown functions in atherosclerosis^47^. It contains a sterile alpha motif (SAM) which is one of the most common protein modules found in eukaryotic genomes and exhibits a wide range of different functions^48,49^. Samd1 has been reported to be an LDL binding protein^47^. Our western blot and qRT-PCR analysis revealed that introduction of miR-378c dramatically decreased while inhibition of miR-378c increased the expression of Samd1 in VSMCs (Fig. 3C,E). Bioinformatic analysis showed that there is a potential miR-378c binding fragment in the 3′UTR of Samd1 gene (Fig. 3F). To further confirm the direct binding of Samd1 by miR-378c, we performed luciferase assay by cloning miR-378c binding fragment (Samd1-3′UTR) as well as its mutated form (Samd1-3′UTR-mut) into a luciferase expressing reporter vector and transfecting miR-378c. We found that forced expression of miR-378c significantly suppressed the activity of the luciferase reporter containing the wild-type 3’UTR of Samd1 but not the mutated form (Fig. 3G), demonstrating the direct binding of Samd1 by miR-378c. Together, these data revealed that Samd1 is a direct target of miR-378. Consistently, blood Samd1 protein level exhibited an inverse correlation with miR-378c (r = − 0.4771, p < 0.01, Fig. 3H).

### Samd1 is essential for miR-378c-mediated VSMCs phenotypic modulation

**Figure 4:**
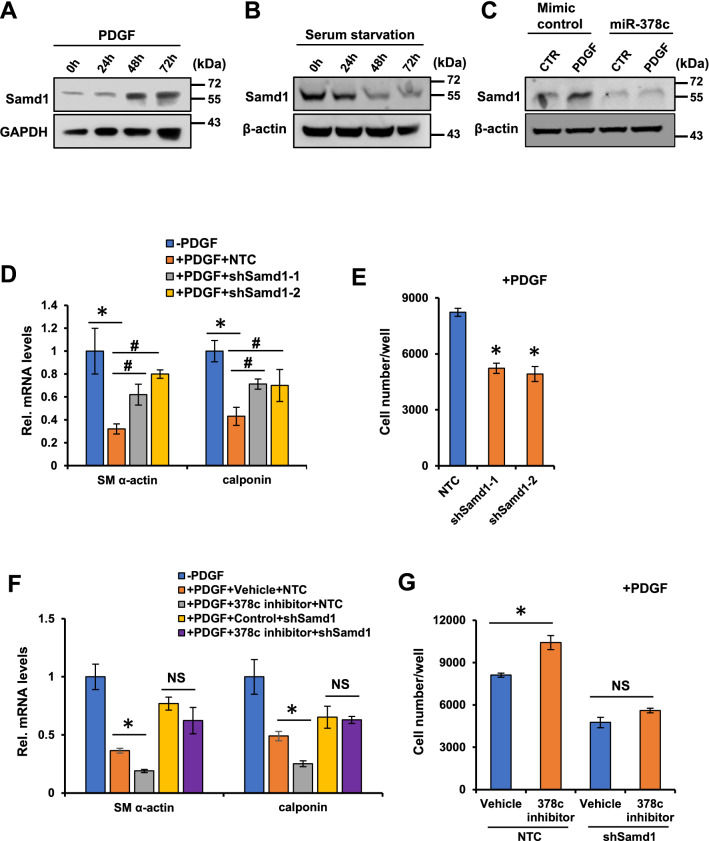
miR-378 controls VSMCs phenotypic modulation thought Samd1. (**A**) Western blot analysis of Samd1 expression in VSMCs stimulated with PDGF-BB. β-actin serves as a loading control. (**B**) Western blot analysis of Samd1 expression in VSMCs stimulated with serum-starvation (0.1%). β-actin serves as a loading control. (**C**) VSMCs stimulated with PDGF-BB were further transfected with miR-378c mimic, followed by western blot analysis of Samd1 expression. β-actin serves as a loading control. (**D**) VSMCs stimulated with PDGF-BB were further transfected with Samd1 shRNA or NTC, VSMCs differentiation markers were analyzed by qRT-PCR. Data were presented as mean ± SD, n = 4. *P < 0.05 as compared with non-PDGF treated group; ^#^P < 0.05 as compared with PDGF-treated NTC group. (**E**) PDGF-BB stimulated VSMCs were further transfected with Samd1 shRNA or NTC, cell numbers were determined. Data were presented as mean ± SD, n = 4. *P < 0.05 as compared with PDGF-treated NTC group. (**F**) PDGF-BB stimulated VSMCs treated with miR-378c inhibitor were further transfected with Samd1 shRNA or NTC, followed by qRT-PCR analysis of VSMC differentiation markers. Data were presented as mean ± SD, n = 4. *P < 0.05 as compared between the indicated group. (**G**) PDGF-BB stimulated VSMCs treated with miR-378c inhibitor were further transfected with Samd1 shRNA or NTC, cell numbers were determined. Data were presented as mean ± SD, n = 4. *P < 0.05 as compared between the indicated group.

Next, we sought to test whether miR-378c regulates VSMC phenotypic modulation through suppressing the expression of Samd1. Western blot analysis showed that Samd1 protein level was markedly stimulated by VSMCs dedifferentiation and suppressed by VSMCs differentiation (Fig. 4A,B). Moreover, PDGF induction of Samd1 expression in VSMCs was completely abolished by the transfection of miR-378c mimic (Fig. 4C), suggesting that miR-378c suppresses phenotypic transition of VSMCs through inhibiting Samd1. Further, knockdown of Samd1 by shRNAs (Supplementary Fig. S2) significantly restored the expression of VSMC differentiation markers suppressed by PDGF in VSMCs (Fig. 4D). Knockdown of Samd1 also suppressed the proliferation of VSMCs (Fig. 4E), demonstrating that Samd1 is involved in VSMCs phenotypic modulation. In addition, knockdown of Samd1 abolished VSMCs phenotypic modulation and VSMCs growth regulated by miR-378c inhibitor (Fig. 4F,G), providing evidence that Samd1 acts downstream of miR-378c in the regulation of VSMCs phenotypic modulation.

### Identification of Samd1 as a previously unrecognized transcriptional repressor

**Figure 5:**
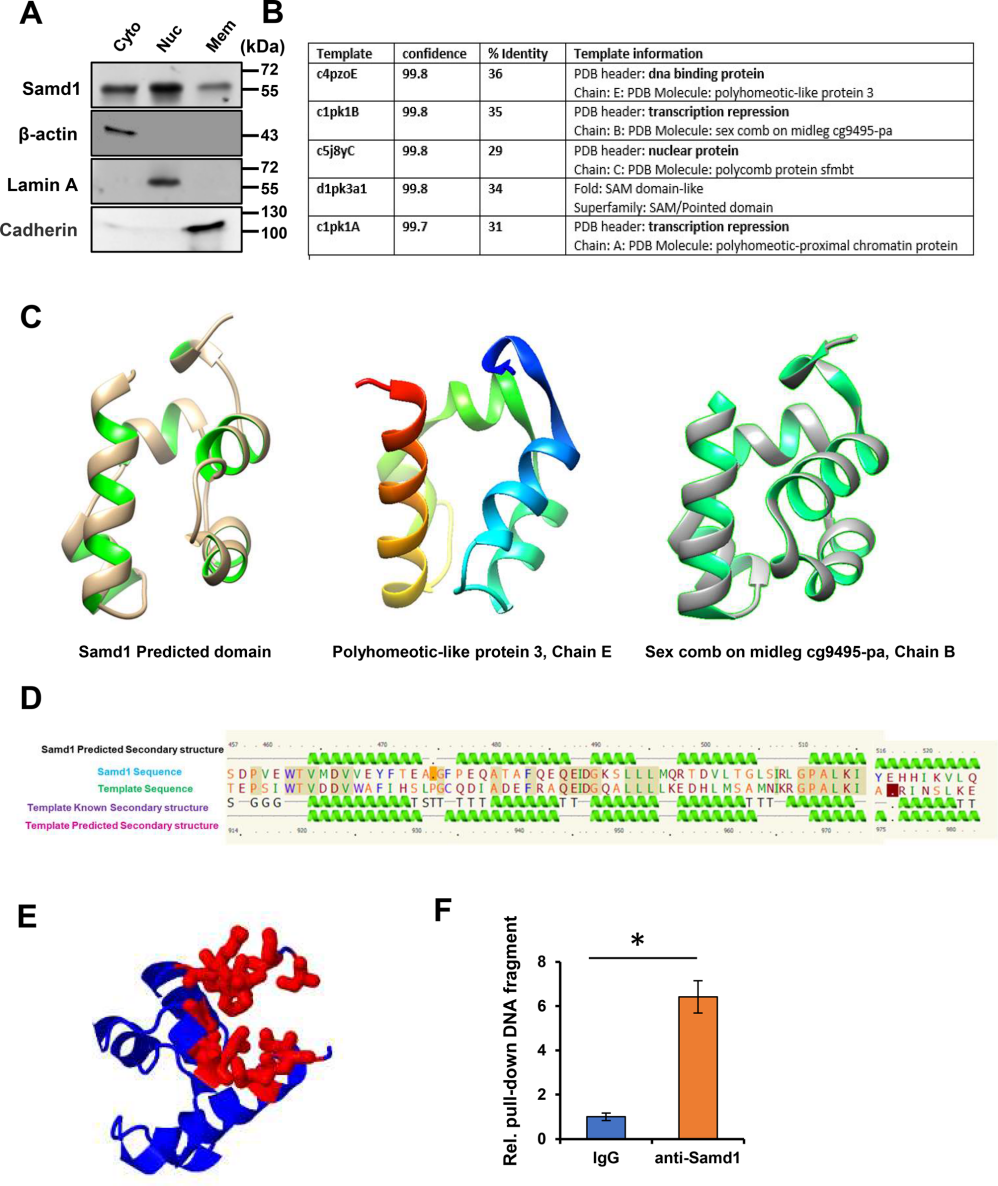
Identification of Samd1 as an unappreciated transcriptional repressor. (**A**) Compartmentalization western blot showing distribution of Samd1 in VSMCs. β-actin serves as cytoplasm marker. Lamin A serves as nuclear marker. Cadherin serves as cell membrane marker. (**B**) Samd1 protein analysis by Phyre2. The Smad1 homologs match has confidence of more than 99.7% and sequence identities of more than 29%. (**C**) Samd1 predicted domain (left); Polyhomeotic-like protein, chain E (modified PDB 4PZO, middle); Sex comb on midleg cg9495-pa, chain B (modified PDB 1PK1, right). (**D**) Samd1 secondary structural predication, comparison, analyzation with known DNA binding protein. (**E**) The pocket detection analysis of Samd1 protein by Fpocket. (**F**) ChIP assay of Samd1 DNA binding using IgG or anti-Samd1 antibody. Data were presented as mean ± SD, n = 4. *P < 0.05 as compared with IgG control.

We further investigated the mechanism by which Samd1 facilitates phenotypic modulation of VSMCs. Intriguingly, compartmentalization western blot revealed an intensive distribution of Samd1 in nucleus (Fig. 5A), which gave us the hint that Samd1 might be involved in transcription regulation.

Sequence alignments phylogenetic analyses, and homology modeling of Samd1 using Phyre2^30^, visualization in UCSF Chimera^31^ helped to validate and evaluate the presence of putative homologs protein domain. Structure analysis of Samd1 showed that it shares a similar secondary structure with some transcriptional repressors^50^ or DNA binding protein domain^51^ (Supplementary Table 3). Even though several various SAM-like domain proteins^52^ were detected in Samd1 as top models (we are not surprising with that since it is SAM domain-containing protein 1), most of the predicted archaeal models contain transcriptional repressor or DNA binding domain. The most promising candidates for Samd1 model were found in transcriptional repressor protein (polyhomeotic-proximal chromatin protein, chain A; sex comb on midleg cg9495-pa, chain B) or DNA binding protein (polyhomeotic-like protein, chain E), both of which are similar as portion of Samd1 (amino acid residues 464–524) with more than 99.7% confidence (Fig. 5B). Three-Dimensional structure comparison of Samd1 motif (Fig. 5C, left) with these two protein domains (Fig. 5C, middle and right) demonstrated that all these three protein domains are very similar as all contain the helix-turn-helix (HTH), a major structural motif capable of binding DNA which are involved in a wide range of functions such as DNA repair, replication^53^ and transcription repression^54^. In addition, secondary structure prediction and comparison of these three protein domains also showing significant similarity between each other, which suggesting Samd1 is a potential transcriptional repressor (Fig. 5D). Consistently, the pocket detection by Fpocket^32^ revealed a major groove through a series of hydrogen bonds and various Van der Waals between the two helix which may play essential roles in DNA interaction (Fig. 5E), and the predicted Samd1 domain is highly conserved by detecting the mutational sensitivity^34^ (Supplementary Fig. S3A). More interestingly, many Samd1 predicted partners also show involvement in the transcription regulation^33^ (Supplementary Fig. S3B).

To further assess whether Samd1 was able to directly bind to DNA, we performed ChIP assay and the results showed a strong binding ability of Samd1 to DNA (Fig. 5F). Together, these findings supported that Samd1 is a potential transcription repressor that probably controls VSMC phenotypic modulation on transcriptional level.

### Samd1 facilitates LDL oxidation and foam cell formation

**Figure 6:**
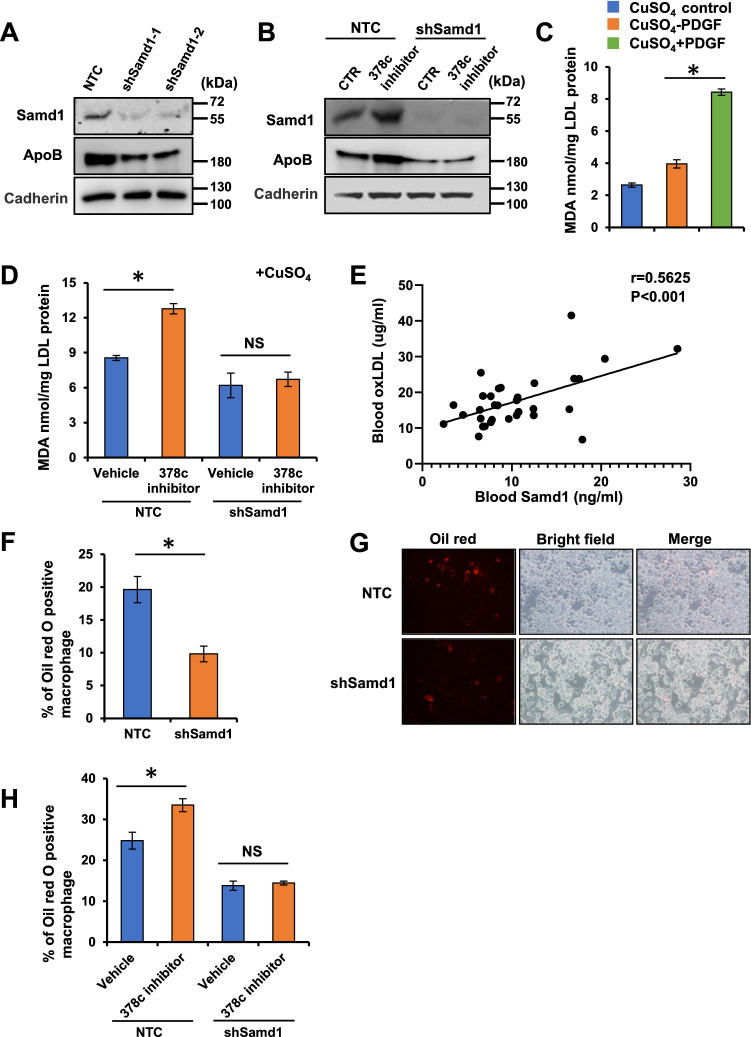
Samd1 facilitates LDL oxidation and foam cell formation. (**A**) VSMCs stimulated with PDGF-BB were further transfected with Samd1 shRNA or NTC and cultured with LDL, membrane Samd1 and ApoB were analyzed by western blot. Cadherin serves as a loading control. (**B**) VSMCs treated with PDGF-BB and miR-378c inhibitor were further transfected with Samd1 shRNA or NTC and cultured with LDL, membrane Samd1 and ApoB were analyzed by western blot. Cadherin serves as a loading control. (**C**) VSMCs were stimulated with PDGF-BB and cultured with LDL. TBARS assay showing oxLDL formation. Data were presented as mean ± SD, n = 4. *P < 0.05 as compared with non-PDGF treated group. (**D**) VSMCs treated with PDGF-BB and miR-378c inhibitor were further transfected with Samd1 shRNA or NTC and cultured with LDL, followed by TBARS assay analysis of oxLDL formation. Data were presented as mean ± SD, n = 4. *P < 0.05 as compared between the indicated group. (**E**) Correlation between blood oxLDL level and blood Samd1 protein level. n = 30. Statistical significance was determined by the chi-square test. (**F**,**G**) VSMCs stimulated with PDGF-BB were further transfected with Samd1 shRNA or NTC and cultured with LDL, supernatants were collected and added to mouse bone-marrow-derived macrophages (BMDM) to induce foam cells formation. Oil red positive cells were counted. Data were presented as mean ± SD, n = 4. *P < 0.05 as compared PDGF-treated NTC group. (**H**) Supernatants from panel (**E**) were collected and added to BMDM to induce foam cells formation. Oil red positive cells were counted. Data were presented as mean ± SD, n = 4. *P < 0.05 as compared between the indicated group.

Samd1 was initially identified as an LDL-binding protein which co-localized with LDL in the progression of atherosclerotic lesions. Prolonged retention of LDL in atherosclerosis-susceptible sites mediated by tightness binding of LDL to arterial wall protein and proteoglycans promotes LDL oxidation, which has a key role in foam cell development and atherogenesis^55–57^. Structure analysis and further compartmentalization western blot result showed that a substantial amount of Samd1 located in the outside surface of cell membrane (Fig. 5A, Supplementary Fig. S4A). We then investigated whether Samd1 could promote the retention of LDL on cell surface. Depletion of Samd1 with shRNAs significantly reduced cell surface Samd1 and ApoB (Fig. 6A). Furthermore, transfection of miR-378c inhibitor caused an increase of cell surface Samd1 and LDL which was further abolished by Samd1 depletion (Fig. 6B), indicating that Samd1 enhanced the retention of LDL on cell surface.

Next, we sought to test whether binding of LDL to Samd1 on cell surface could facilitate the oxidation of LDL. Cells in the arterial intima can promote LDL oxidation by its enzymes^58^, oxidation of LDL leads to the production of malondialdehyde (MDA) which could be measured by reaction with TBA with slight modification^29,59,60^. Next, TBARS assay showed that TBARS formation was significantly increased in the supernatant of VSMCs following PDGF stimulation (Fig. 6C). More importantly, inhibition of miR-378c increased the formation of TBARS, which was further blocked by Samd1 knockdown (Fig. 6D). Next, we evaluated the physiological relevance of circulating oxidized LDL (oxLDL) level and Samd1 protein abundance in vivo. Statistical analysis using a cohort of blood samples from 30 patient revealed a striking positive correlation between Samd1 protein level and oxidized LDL level (Fig. 6E). Together, these results indicated that Samd1 promotes LDL oxidation by binding with LDL on cell surface.

Oxidized LDL plays a critical role in atherosclerosis through its involvement in the formation of foam cells. After uptaking oxLDL, macrophages can differentiate into foam cells and produce diverse growth factors and proinflammatory cytokines such as tumor necrosis factor (TNF)-a and interleukins (ILs). These factors further stimulate the proliferation of VSMCs and lead to plaque formation^61,62^. Supernatant from VSMCs with Samd1 knockdown significantly reduced the formation of foam cells as indicated by oil red stain and secretion of proinflammatory cytokines (Fig. 6F, 6G, Supplementary Fig. S4B). Meanwhile, supernatant from VSMCs pre-treated with miR-378c inhibitor promoted the formation of foam cells, which was abrogated by Samd1 knock down (Fig. 6H, Supplementary Fig. S4C). Taken together, our results demonstrated that Samd1 binds and promotes the oxidation of LDL, which prolongs the retention of LDL on VSM cell surface and leads to the formation of foam cell.

## Discussion

**Figure 7:**
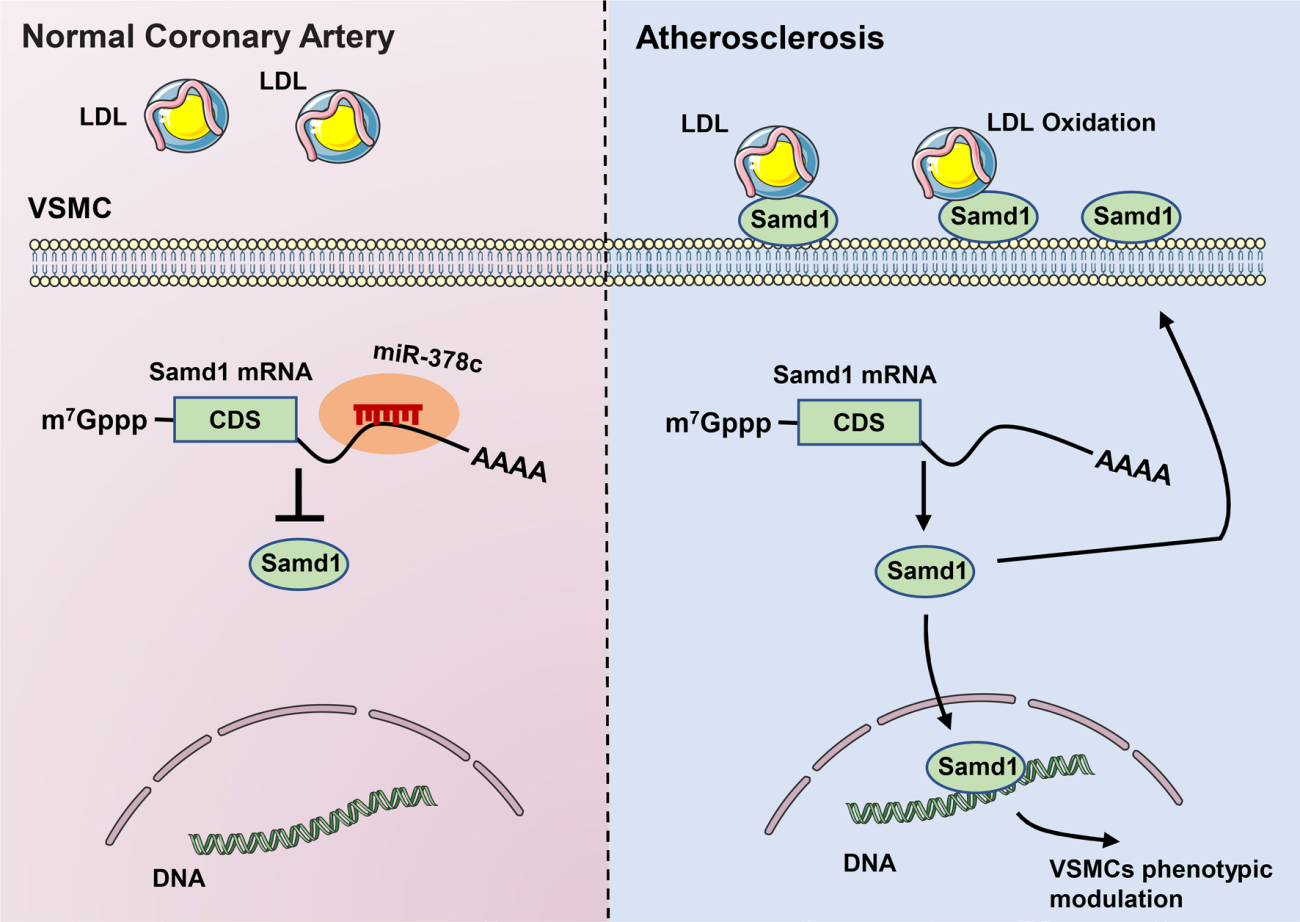
Working model: The miR-378c-Samd1 circuit promotes phenotypic modulation of vascular smooth muscle cells and foam cells formation in atherosclerosis lesions.

Atherosclerosis is a complex multifactorial disease that, despite advances in lifestyle management and drug therapy, remains to be the leading cause of death and disability in the world. Although emerging studies have revealed pathogenesis of atherosclerosis, the exact cause is still unknown. By comparing MicroRNAs profiling of human normal coronary artery and artery with plaques, we identified miR-378c as a major down-regulated MicroRNA in atherosclerosis. Further studies of miR-378c demonstrated that down-regulation of miR-378c expression promotes VSMCs phenotypic modulation, which is an important event in early atherosclerosis. Moreover, tracing the downstream targets of miR-378c led us to find Samd1, a newly identified, potential LDL-binding protein. Interestingly, analysis of Samd1 revealed that Samd1 functions as a transcriptional repressor. Thus, miR-378c repression increased Samd1 level and promoted VSMCs phenotypic modulation. Meanwhile, binding of Samd1 with LDL on cell surface facilitated the oxidation of LDL and subsequently foam cell formation (Fig. 7). Together, our findings uncovered miR-378c as a previously unappreciated factor that plays critical roles in VSMCs phenotypic modulation.

Early detection and diagnosis of atherosclerosis has shown great importance in atherosclerosis treatment and understanding atherosclerosis risk factors in early stage can assist pre-drug treatment and significantly improve patient outcomes^63,64^. Despite advances in diagnostic tools, many patients at increased risk for acute coronary events are not identified, as they do not present with any symptoms prior to an acute coronary event. Hence, novel diagnostic markers are needed to better assess a patients’ risk of atherosclerosis. The recent emergence of MicroRNAs as important regulators of atherosclerosis has provided novel molecular insights into pathogenesis of atherosclerosis and new therapeutic targets^65,66^. In addition, the appreciation that MicroRNAs can be detected extracellularly including in circulating blood raises the potential for their use as biomarkers for early diagnosis and prognosis^67,68^. Here, we identified miR-378c as a promising blood indicator of atherosclerosis. miR-378 is closely related to cancer and other diseases, aberrant miR-378 expression has been observed in various types of cancer. In addition, the involvement of miR-378 in heart diseases and diabetes has also been reported. Using microarray, we discovered that miR-378c was significantly suppressed in atherosclerotic plaques. Further study demonstrated that down-regulation of miR-378c promoted VSMCs phenotypic modulation and proliferation and facilitated foam cell formation through targeting Samd1. Since suppressed miR-378c expression was found at all stages of atherosclerotic lesions, together with its function in atherosclerosis, miR-378c was found to be a novel regulator of atherosclerosis initiation and a biomarker for early atherosclerosis detection.

It is very intriguing that we identified Samd1 as a previously unrecognized transcriptional repressor that plays a vital role in the development of atherosclerosis. Samd1 is a newly identified protein with little known function. In our study we demonstrated that the presumptive DNA-binding domain of Samd1 has sequence similarity to some transcriptional repressor^50^ or DNA binding protein domain^51^. The most promising candidates for Samd1 were found in transcriptional repressor protein or DNA binding protein, both of which are more than 99.7% similarity as portion of Samd1. A transcription repressor blocks the connection of RNA polymerase to the promoter, consequently preventing transcription. Samd1 partners was unexpectedly found to be involved in DNA transcription. Among them, L3MBTL3 maintains the transcriptionally repressive state of genes^69,70^; ELP3 is a component of the RNA polymerase II (Pol II) holoenzyme and is involved in transcriptional elongation^71,72^; EPC1 is involved in transcriptional activation of select genes^73^; EPC2 plays a role in transcription or DNA repair^74^ etc. This finding also coincides with our hypothesis that Smad1 is a potential transcription repressor. Identification of Samd1 as the potential regulator of transcriptional responses has far-reaching implications. The precise control of gene expression levels is known to be important for determining cell fate. For any given cell type, distinct transcriptional programs must be established whereby certain genes are transcribed and others remain silent, which highlights the important involvement of activators or repressors in transcription regulation^75,76^. Meanwhile, dysregulation of activators or repressors expression is tightly associated with multiple diseases, such as cancer^77,78^. For this regard, discovering Samd1 as a potential transcriptional repressor will provide new insight into understanding gene expression control and the development of related diseases. Hence, elucidation of the functional mechanism of Samd1 as well as specific function in transcriptional regulation are needed in further investigations.

A critical challenge for future atherosclerosis studies will be to develop effective treatment approaches. Delivery of a cassette of MicroRNA mimics or inhibitors may thereby offer a more promising therapeutic approach to facilitate fine-tuning specific stages of atherosclerotic diseases. Collectively, our finding provides a potential rationale to target miR-378c-Samd1 pathway for the detection and treatment of atherosclerosis.

### Limitations of study

Our studies were conducted in cultured cells (in vitro) and whether comparable effects will be observed murine models (in vivo*)* remains to be assessed, although VSMCs play important roles in atherosclerosis. Future studies will include the murine models of miR-378 knockout to validate the pathway proposed in the study and the identification of the targets of Samd1.

## Supplementary Information

Supplementary Information 1.

Supplementary Information 2.

Supplementary Information 3.

Supplementary Information 4.

Supplementary Information 5.

Supplementary Information 6.
