# Supplementary material for: The miR-378c-Samd1 circuit promotes phenotypic modulation of vascular smooth muscle cells and foam cells formation in atherosclerosis lesions
Source: Sci Rep. 2021 May 18;11:10548. doi: 10.1038/s41598-021-89981-z (PMC8131603; doi:10.1038/s41598-021-89981-z)
Supplement: Supplementary file 1 — Supplementary Information 1. [file 41598_2021_89981_MOESM1_ESM.pdf]

The miR-378c-Samd1 Circuit Promotes Phenotypic Modulation of Vascular Smooth Muscle Cells and Foam Cells Formation in Atherosclerosis Lesions

Shengya Tian[3*], Yang Cao[4*], JinLiang Wang[5*], Yongjun Bi[6], Jingquan Zhong[1], Xiangbin Meng[7], Wenyu Sun[8], Ruixue Yang[1], Luping Gan[9], Xuping Wang[1], Hongshi Li[1], Rong Wang[1,2]

[1]The Key Laboratory of Cardiovascular Remodeling and Function Research, Chinese Ministry of Education, Chinese National Health Commission and Chinese Academy of Medical Sciences, The State and Shandong Province Joint Key Laboratory of Translational Cardiovascular Medicine, Department of Cardiology, Qilu Hospital, Cheeloo College of Medicine, Shandong University, 107 Wenhuaxi Road, Jinan, Shandong 250012, China

[2]Department of Cardiology, Boston Children's Hospital, Harvard Medical School, 300 Longwood Avenue, Boston, MA 02115, USA.

[3]Division of Hematology and Oncology, Beth Israel Deaconess Medical Center (BIDMC) and Harvard Medical School (HMS), Boston, MA 02215, USA

[4]Department of Medicine/Division of Cardiology, University of California, Los Angeles, CA, USA

[5]Department of Biochemistry, University of Utah School of Medicine, Salt Lake City, UT 84112, USA

[6]Clinical Laboratory, Central Hospital of Xinwen Mining Group, 164 Xinkuang Road, Xintai, Shandong 271233, China

[7]Department of Cardiac Surgery, Qilu Hospital, Cheeloo College of Medicine, Shandong University, 107 Wenhuaxi Road, Jinan, Shandong 250012, China

[8]Department of Cardiac Surgery, Qilu Hospital (Qingdao), Cheeloo College of Medicine, Shandong University, 758 Hefei Road, Qingdao, Shandong, 266035, China

[9]Department of Anesthesiology, The People's Hospital of Yichun Jiangxi, Yichun, Jiangxi 336000, China

*These authors contributed equally to this work.

Correspondence: Rong Wang

E-mail: rongw214573@yahoo.com

Supplemental figure legends

Figure S1. Association of blood vessel lesions with FBG, HDL, LDL and TC

FBG, fasting blood glucose; HDL, high-density lipoprotein; LDL, low-density lipoprotein; TC, total cholesterol.

Figure S2. Samd1 knockdown by shRNAs

Western blot analysis of Samd1 knock down efficiency in VSMCs. β-actin serves as a loading control.

Figure S3. Prediction of Samd1 as a transcriptional repressor

(A) The mutational sensitivity analysis by SuSPect. Distribution of SuSPect for highly sensitive (red) and neutral (blue) Amino Acid Variant (SAV) in the Samd1 predicted domain.

(B) Samd1 predicted binding partners obtained, visualized, and modified by STRING v10.

Figure S4. Samd1 facilitates LDL oxidation and foam cell formation

(A) TMHMM analysis showing outside membrane distribution of Samd1

(B) VSMCs stimulated with PDGF-BB were further transfected with Samd1 shRNA or NTC and culture with LDL, supernatants were collected and adding to mouse bone-marrow-derived macrophages (BMDM) to induce foam cells formation. Secretion of TNF-α and IL-6 were analyzed. Data were presented as mean ± SD. *P < 0.05 as compared PDGF-treated NTC group.

(C) VSMCs treated with PDGF-BB and miR-378c inhibitor were further transfected with Samd1 shRNA or NTC and cultured with LDL, supernatants were collected and added to mouse bone-marrow-derived macrophages (BMDM) to induce foam cells formation. Secretion of TNF-α and IL-6 were analyzed. Data were presented as mean ± SD. *P < 0.05 as compared between the indicated group.

Table legends

Table 1. MicroRNA array. human artery with plaques versus normal coronary artery

Table 2. MicroRNAs that were significantly up-regulated and down-regulated in artery with plaques

Table 3. Threading template identification for Samd1 using profile-profile alignments by Phyre2

Table 4. Nucleotide Sequence of Primers Used for qRT-PCR

Table 5. Clinical human specimen information

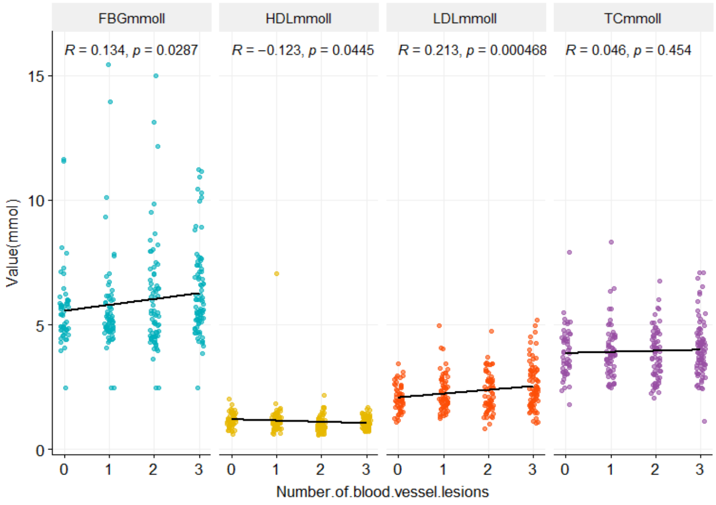

Figure S1. Association of blood vessel lesions with FBG, HDL, LDL and TC

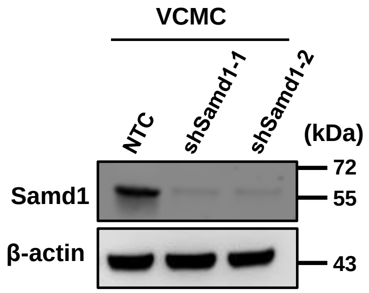

Figure S2. Samd1 knockdown by shRNAs

A

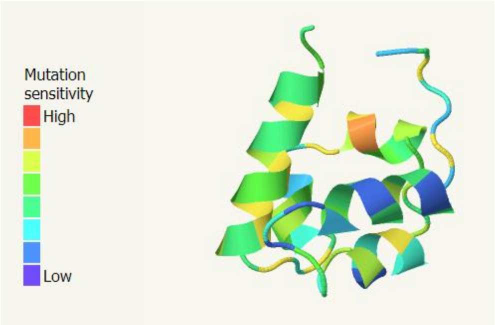

B

Predicted Functional Partners

Predicted Partners	Function
L3MBTL3	maintain the transcriptionally repressive state of genes
MSL1	Component of histone acetyltransferase complex responsible for the majority of histone H4 acetylation at 'Lys-16' (H4K16ac) which is implicated in the formation of higher-order chromatin structure.
RNF222	Ring finger protein
PRMT2	Protein arginine N-methyltransferase 2
ELP3	Elongator complex protein 3; Catalytic histone acetyltransferase subunit of the RNA polymerase II elongator complex, which is a component of the RNA polymerase II (Pol II) holoenzyme and is involved in transcriptional elongation.
EPC1	Enhancer of polycomb homolog 1; Component of the NuA4 histone acetyltransferase (HAT) complex which is involved in transcriptional activation of select genes principally by acetylation of nucleosomal histones H4 and H2A
EPC2	Enhancer of polycomb homolog2; May play a role in transcription or DNA repair

Figure S3. Prediction of Samd1 as a transcriptional repressor

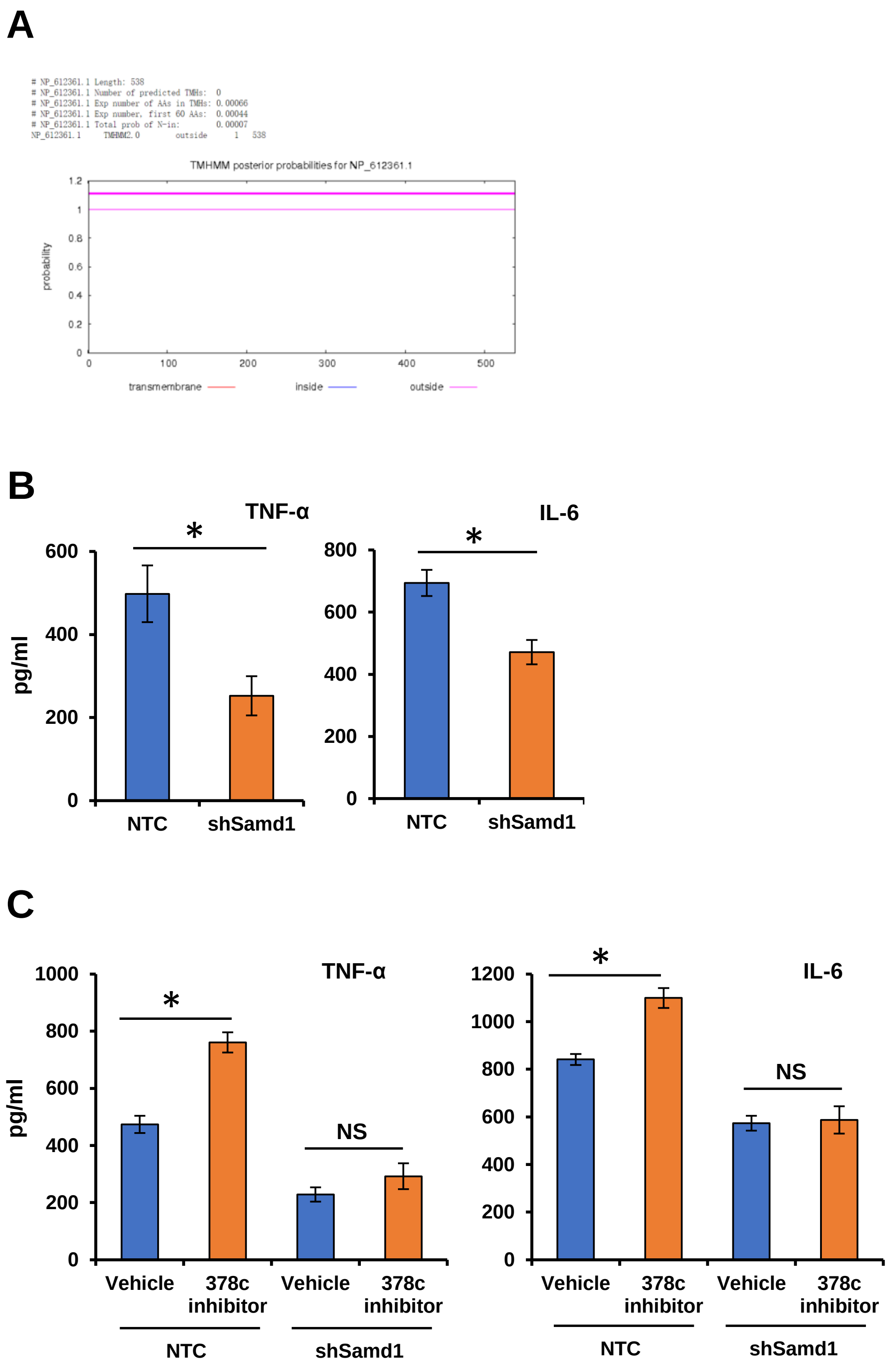

Figure S4. Samd1 facilitates LDL oxidation and foam cell formation

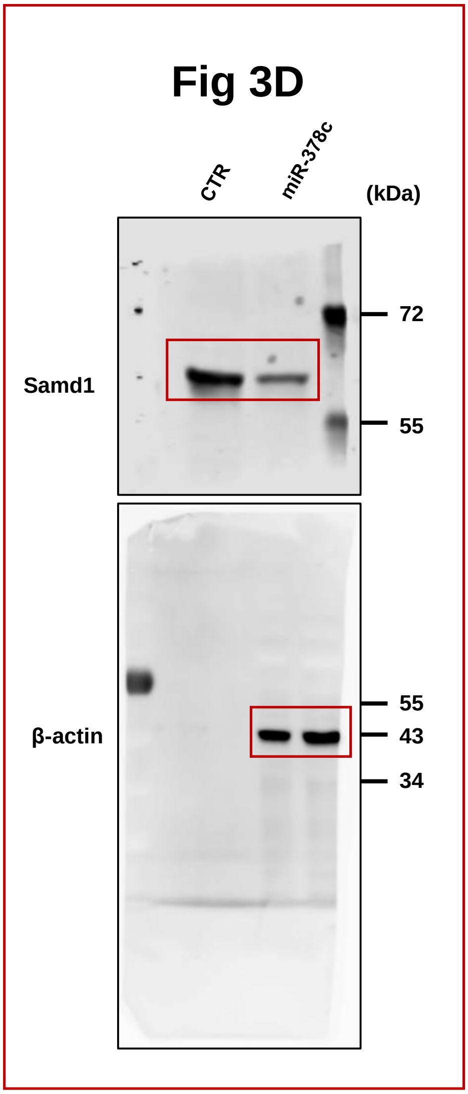

Figure 3. uncropped, unedited

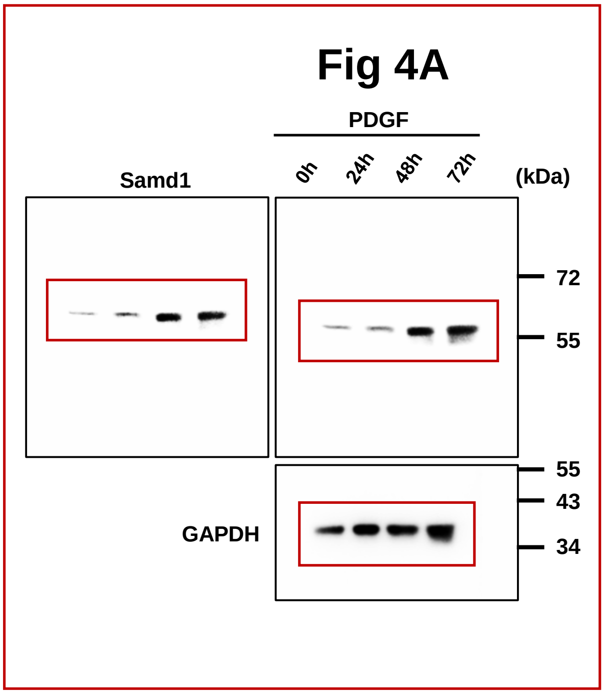

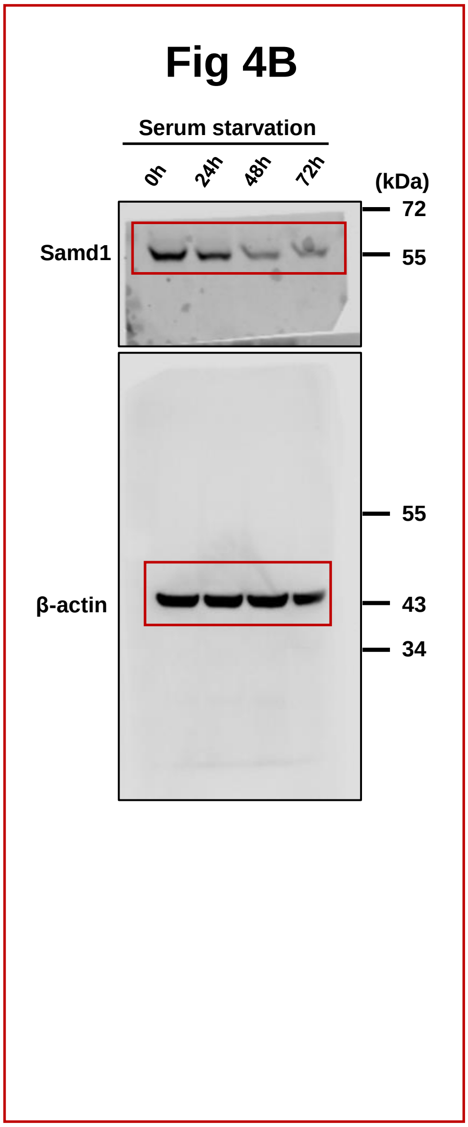

Figure 4. uncropped, unedited

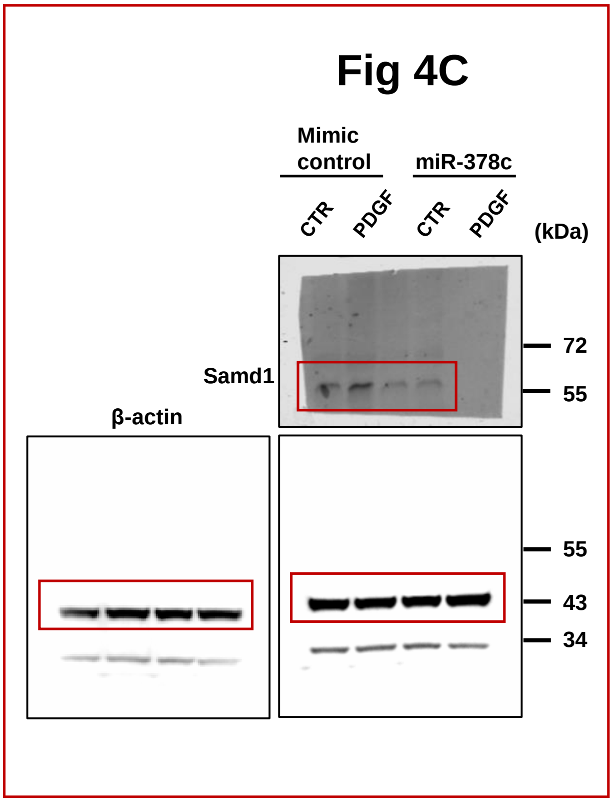

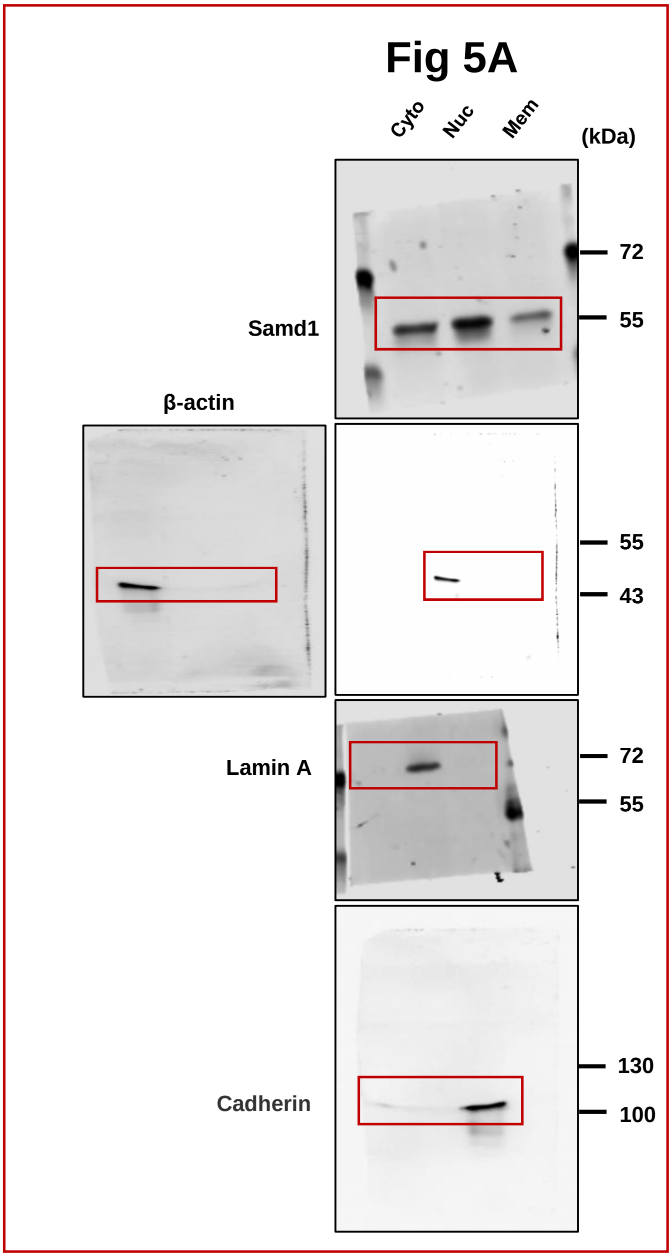

Figure 4-5. uncropped, unedited

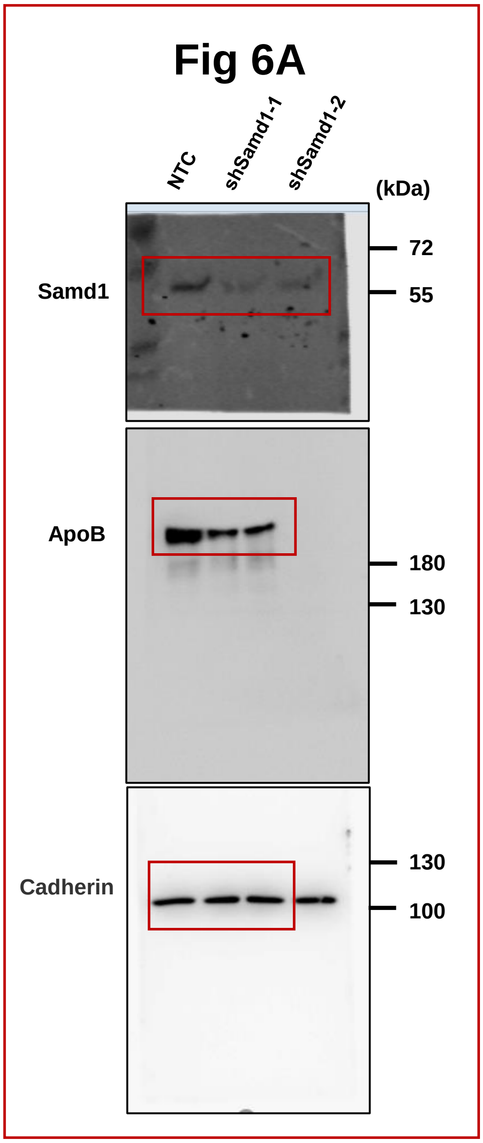

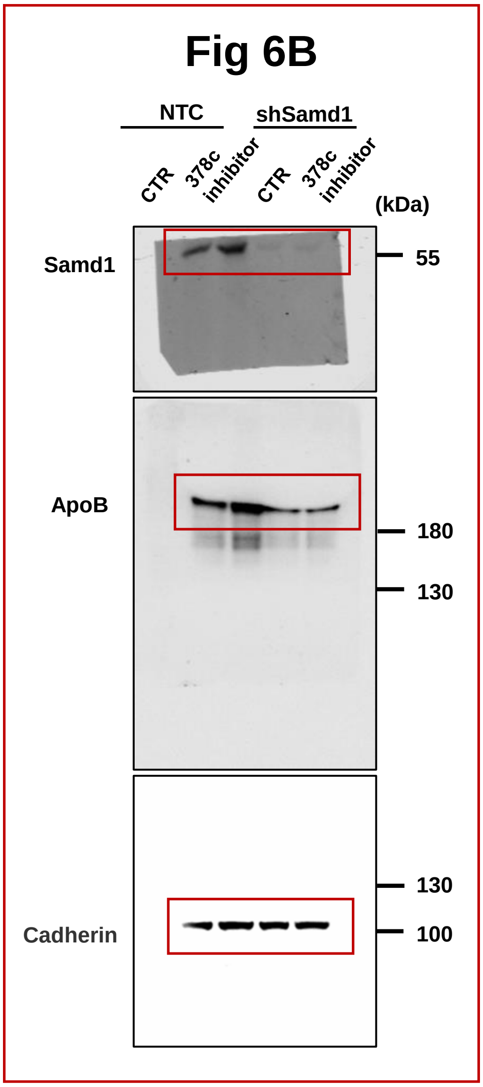

Figure 6. uncropped, unedited

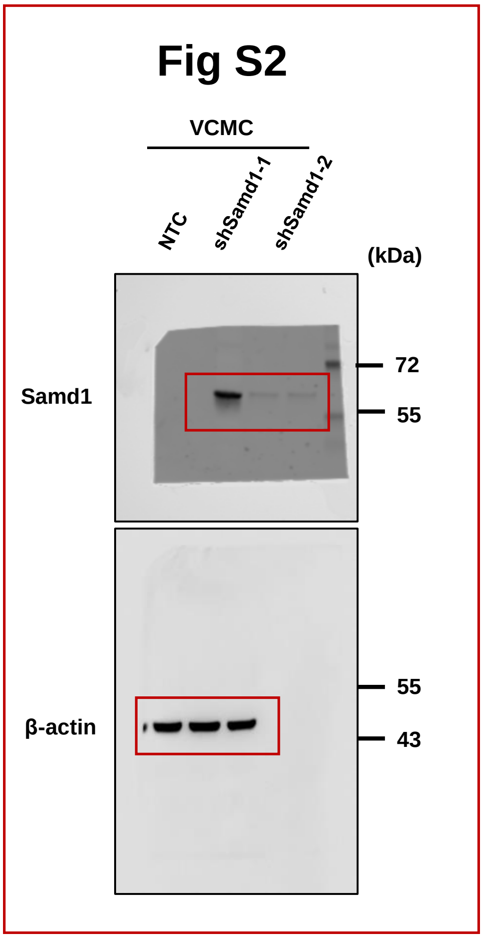

Figure S2. uncropped, unedited
