# Supplementary material for: The miR-378c-Samd1 circuit promotes phenotypic modulation of vascular smooth muscle cells and foam cells formation in atherosclerosis lesions
Source: Sci Rep. 2021 May 18;11:10548. doi: 10.1038/s41598-021-89981-z (PMC8131603; doi:10.1038/s41598-021-89981-z)
Supplement: Supplementary file 4 — Supplementary Information 4. [file 41598_2021_89981_MOESM4_ESM.pdf]

Phyre2

Detailed template information

#	Template	Alignment Coverage	3D Model	Confidence	% i.d.	Template Information
1	c4pzoE_	Alignment	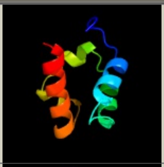	99.8	36	**PDB header:**dna binding protein<br>**Chain:** E: **PDB Molecule:**polyhomeotic-like protein 3;<br>**PDBTitle:** crystal structure of phc3 sam l967r
2	c1pk1B_	Alignment	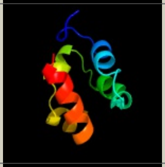	99.8	35	**PDB header:**transcription repression<br>**Chain:** B: **PDB Molecule:**sex comb on midleg cg9495-pa;<br>**PDBTitle:** hetero sam domain structure of ph and scm.
3	c5j8yC_	Alignment	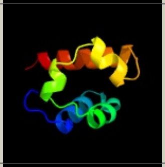	99.8	29	**PDB header:**nuclear protein<br>**Chain:** C: **PDB Molecule:**polycomb protein sfmbt;<br>**PDBTitle:** crystal structure of the scm-sam and sfmbt-sam heterodimer
4	d1pk3a1	Alignment	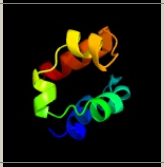	99.8	34	**Fold:**SAM domain-like<br>**Superfamily:**SAM/Pointed domain<br>**Family:**SAM (sterile alpha motif) domain
5	c1pk1A_	Alignment	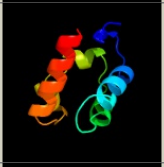	99.7	31	**PDB header:**transcription repression<br>**Chain:** A: **PDB Molecule:**polyhomeotic-proximal chromatin protein;<br>**PDBTitle:** hetero sam domain structure of ph and scm.
6	d1pk1c1	Alignment	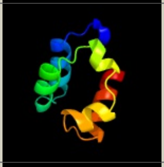	99.7	32	**Fold:**SAM domain-like<br>**Superfamily:**SAM/Pointed domain<br>**Family:**SAM (sterile alpha motif) domain
7	d1kw4a_	Alignment	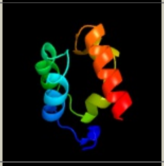	99.7	31	**Fold:**SAM domain-like<br>**Superfamily:**SAM/Pointed domain<br>**Family:**SAM (sterile alpha motif) domain
8	c2dkzA_	Alignment	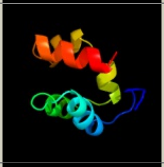	99.7	17	**PDB header:**signaling protein<br>**Chain:** A: **PDB Molecule:**hypothetical protein loc64762;<br>**PDBTitle:** solution structure of the sam_pnt-domain of the2 hypothetical protein loc64762
9	c2e8oA_	Alignment	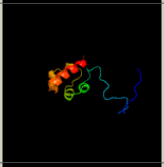	99.7	19	**PDB header:**signaling protein<br>**Chain:** A: **PDB Molecule:**sam domain and hd domain-containing protein 1;<br>**PDBTitle:** solution structure of the n-terminal sam-domain of the sam2 domain and hd domain containing protein 1 (dendritic cell-3 derived ifng-induced protein) (dcip) (monocyte protein 5)4 (mop-5)
10	d1uqva_	Alignment	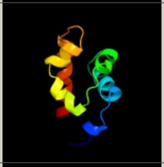	99.6	19	**Fold:**SAM domain-like<br>**Superfamily:**SAM/Pointed domain<br>**Family:**SAM (sterile alpha motif) domain
11	d1wwva1	Alignment	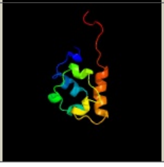	99.5	19	**Fold:**SAM domain-like<br>**Superfamily:**SAM/Pointed domain<br>**Family:**SAM (sterile alpha motif) domain

12	c1v85A_	Alignment	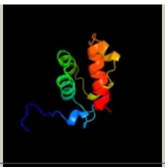	99.5	16	**PDB header:**apoptosis<br>**Chain:** A: **PDB Molecule:**similar to ring finger protein 36;<br>**PDBTitle:** sterile alpha motif (sam) domain of mouse bifunctional2 apoptosis regulator
13	c2eanA_	Alignment	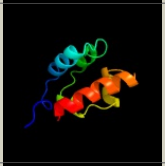	99.5	22	**PDB header:**signaling protein<br>**Chain:** A: **PDB Molecule:**connector enhancer of kinase suppressor of ras 2;<br>**PDBTitle:** solution structure of the n-terminal sam-domain of human2 kiaa0902 protein (connector enhancer of kinase suppressor3 of ras 2)
14	c3bs5A_	Alignment	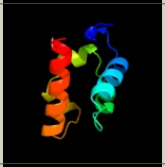	99.5	19	**PDB header:**signaling protein/membrane protein<br>**Chain:** A: **PDB Molecule:**protein aveugle;<br>**PDBTitle:** crystal structure of hcnk2-sam/dhyp-sam complex
15	c3bq7A_	Alignment	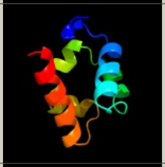	99.5	21	**PDB header:**transferase<br>**Chain:** A: **PDB Molecule:**diacylglycerol kinase delta;<br>**PDBTitle:** sam domain of diacylglycerol kinase delta1 (e35g)
16	c2gleA_	Alignment	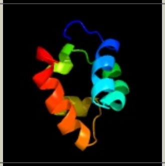	99.5	17	**PDB header:**protein binding<br>**Chain:** A: **PDB Molecule:**neurabin-1;<br>**PDBTitle:** solution structure of neurabin sam domain
17	c6qwvN_	Alignment	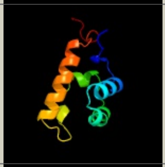	99.5	17	**PDB header:**apoptosis<br>**Chain:** N: **PDB Molecule:**sterile alpha and tir motif-containing protein 1;<br>**PDBTitle:** sarm1 sam1-2 domains
18	d2f3na1	Alignment	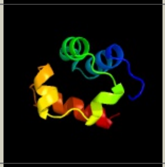	99.4	22	**Fold:**SAM domain-like<br>**Superfamily:**SAM/Pointed domain<br>**Family:**SAM (sterile alpha motif) domain
19	c3bs7A_	Alignment	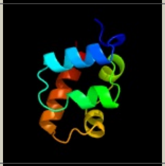	99.4	19	**PDB header:**signaling protein<br>**Chain:** A: **PDB Molecule:**protein aveugle;<br>**PDBTitle:** crystal structure of the sterile alpha motif (sam) domain2 of hyphen/aveugle
20	c2l5yA_	Alignment	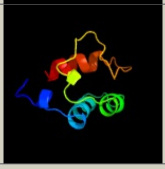	99.4	20	**PDB header:**signaling protein<br>**Chain:** A: **PDB Molecule:**stromal interaction molecule 2;<br>**PDBTitle:** nmr structure of calcium-loaded stim2 ef-sam.
21	c5f3xB_	Alignment	not modelled	99.3	23	**PDB header:**structural protein/protein binding<br>**Chain:** B: **PDB Molecule:**ankyrin repeat and sam domain-containing protein 4b;<br>**PDBTitle:** crystal structure of harmonin npdz1 in complex with anks4b sam-pbm
22	c6pw7B_	Alignment	not modelled	99.3	24	**PDB header:**signaling protein<br>**Chain:** B: **PDB Molecule:**stromal interaction molecule 1;<br>**PDBTitle:** x-ray crystal structure of c. elegans stim ef-sam domain
23	c3k1rB_	Alignment	not modelled	99.3	24	**PDB header:**structural protein<br>**Chain:** B: **PDB Molecule:**usher syndrome type-1g protein;<br>**PDBTitle:** structure of harmonin npdz1 in complex with the sam-pbm of2 sans
24	c2k60A_	Alignment	not modelled	99.3	20	**PDB header:**signaling protein<br>**Chain:** A: **PDB Molecule:**protein (stromal interaction molecule 1);<br>**PDBTitle:** nmr structure of calcium-loaded stim1 ef-sam
25	c2eaoA_	Alignment	not modelled	99.3	16	**PDB header:**signaling protein, transferase<br>**Chain:** A: **PDB Molecule:**ephrin type-b receptor 1;<br>**PDBTitle:** solution structure of the c-terminal sam-domain of mouse2 ephrin type-b receptor 1 precursor (ec 2.7.1.112)
26	c6kr4H_	Alignment	not modelled	99.3	15	**PDB header:**transferase/protein binding<br>**Chain:** H: **PDB Molecule:**liprin-alpha-3;<br>**PDBTitle:** crystal structure of the liprin-alpha3_sam123/lar_d1d2 complex
27	c3tadB_	Alignment	not modelled	99.3	13	**PDB header:**protein binding<br>**Chain:** B: **PDB Molecule:**liprin-alpha-2;<br>**PDBTitle:** crystal structure of the liprin-alpha/liprin-beta complex
28	c5jtiA_	Alignment	not modelled	99.2	17	**PDB header:**signaling protein<br>**Chain:** A: **PDB Molecule:**tankyrase-1;<br>**PDBTitle:** crystal structure of the human tankyrase 1 (tnks) sam domain (d1055r),2 crystal form 2
